# Protective factors against disordered eating in family systems: a systematic review of research

**DOI:** 10.1186/s40337-017-0141-7

**Published:** 2017-03-28

**Authors:** Jasmin Langdon-Daly, Lucy Serpell

**Affiliations:** Department of Clinical, Educational, and Health Psychology, University College London, London, UK

**Keywords:** Eating disorders, Disordered eating, Family, Protective factors, Systematic review

## Abstract

**Objective:**

This systematic review aims to identify and evaluate the literature investigating protective factors and eating disorders (EDs), to establish what is known about factors in family systems that could be considered protective against the development of ED/disordered eating.

**Methods:**

A systematic review of the literature was conducted on five databases, using search terms related to ED/disordered eating and protective factors. Studies were systematically screened and included if they made reference to a protective factor within the family system and explored associations with a quantitative measure of ED/disordered eating behaviours. All included studies were evaluated for study quality.

**Results:**

Twenty-five studies met criteria for inclusion. Ten papers made use of longitudinal or prospective designs appropriate to identify factors potentially protecting against the development of disordered eating difficulties, while a further 15 papers report cross-sectional associations between family factors and disordered eating outcomes. Studies looked at aspects of family relationships and family practices around food or eating. There was a particular research focus on the potential protective role of regular family meals.

**Conclusions and Implications:**

Many of the potential protective factors identified, such as family support and connectedness, may be non-specific to eating difficulties, promoting general adaptive development and a range of positive development outcomes. Factors in the family environment around food, eating and weight, such as frequent family meals and avoiding comments about weight, may be more specific to ED and disordered eating. Issues with the methodologies used severely impact on the ability to draw conclusions about whether factors are ‘protective’.

## Plain English Summary

This paper is a review of research studying characteristics of families which make it less likely for young people to develop problems with eating and eating disorders. These characteristics are known as ‘protective factors’. We systematically reviewed the scientific literature, using a fixed set of search terms to search databases of scientific journal articles, and then selecting the 25 studies meeting our criteria. We report the methods used in the studies and the key research findings. The research papers discussed identify lots of possible ‘protective factors’. Having a healthy family environment around eating (for example having frequent family meals and avoiding comments about weight) and other characteristics like high quality family relationships, may help to protect young people against problems with eating. We also talk about some of the strengths and weaknesses of the methods used in the different studies. Some of these weaknesses mean that we can’t be certain if some of the studies really did identify a ‘protective factor’. We suggest that looking at protective factors in families might be helpful to people who are trying to protect young people from eating problems, and make suggestions for further research needed to find out more about these issues.

## Background

Eating disorders (ED), including anorexia nervosa (AN), bulimia nervosa (BN), binge-eating disorder (BED), and other eating difficulties causing clinically significant levels of distress and impairment, are serious mental health conditions associated with a range of negative physical, psychological and social outcomes [1]. Sub-clinical ‘disordered eating’ or ‘eating pathology’ affects a large proportion of the population, with some studies finding that up to 20% of young women report the use of disordered eating behaviours such as using diet pills, vomiting or laxatives to manage their weight [2]. These difficulties therefore represent a major public health concern, and there is growing recognition of the need to understand the factors influencing their development in order to inform efforts to prevent them. A focus on protective factors and building strengths, rather than on reducing not fully developed risks, may be particularly useful in designing universal prevention programmes for young people [3].

Theorists and clinicians working in the EDs field have highlighted the importance of considering and working with the family system when attempting to understand and intervene with these difficulties [4]. Families are viewed as an invaluable resource to promote recovery in many interventions for ED [5]. Large numbers of studies have aimed to elucidate the risk factors for the development of EDs at different levels of influence, and a range of factors within families (such as parental weight related teasing and parental encouragement to diet) have been suggested to increase risk of eating pathology [6–9]. Protective factors also occur across multiple levels of context, including at the level of the family system. Consideration of protective factors at the level of the family system could inform efforts to prevent disordered eating, by promoting processes and practices which protect against those outcomes [6, 10, 11]. Increased awareness of factors in family systems which are protective is likely to be useful not just to clinicians, but also to families, schools and communities hoping promote adaptive development and help to prevent disordered eating difficulties [6].

### ‘Protective’ factors

The term ‘protective factor’ has been defined in different ways dependent on the theoretical framework in use [3, 12]. Within the developmental psychopathology framework, a protective factor is something which moderates the effect of a vulnerability or risk factor on development, promoting adaptive development and ‘resilience’ (the capacity for positive outcomes despite challenging circumstances) [13, 14]. Theorists from the ‘positive psychology’ movement have argued that the dominant paradigm focus on risk factors and pathology has resulted in neglect of strengths and positive aspects of individuals and systems [12, 15]. They encourage the investigation of protective factors, those which promote health and wellbeing in everyone, and suggest that prevention programmes should aim to develop and foster these. These ideas have informed the concept of ‘developmental assets’, a range of contextual (family, peer, school, community) and individual factors which form a set of ‘building blocks’ for successful development and positive outcomes. The presence of these developmental assets is thought to protect against the initiation of a range of health risk behaviours [16]. Indeed, many protective factors are thought to be non-specific, reducing the probability of the onset of a range of difficulties [3]. Despite this, protective factors are not necessarily universal, and what constitutes a protective factor will vary depending on gender, social class, ethnicity, age, and other variables.

Kraemer et al. argue that there is a need for more precision in the definition and use of the terms ‘risk factor’ and ‘protective factor’ [17]. They define protective factors as “factors that either identify subjects at lower risk for the disorder or a higher probability of welcome outcomes”. Key to their definition is the idea of establishing precedence, without which a factor can only be considered a ‘correlate’, and may well be a ‘concomitant’ or a ‘consequence’. They have pointed out that the preponderance of cross-sectional and retrospective designs in the psychiatric literature means that many factors named as ‘risk’ or ‘protective’ factors should in fact only be considered ‘correlates’, limiting their clinical or theoretical utility. Their ‘typography of risk’ also distinguishes between fixed factors (which cannot change), variable risk/protective factors (which vary over time spontaneously or can be modified) and ‘causal risk/protective factors’ (which are manipulable, and change the likelihood of an outcome when modified). They suggest that ‘causal factors’ may be of interest to clinicians, but are clear to differentiate them from a ‘cause’, highlighting the probabilistic nature of outcomes, the likely complex interaction of many factors, and the possibility of multiple pathways to the same outcome.

### Aims

While a number of studies have identified potential protective factors for disordered eating in families, no cohesive picture of research into these factors has been developed and the field has not previously been systematically reviewed. Reviewing this literature could be of relevance to prevention efforts at the family level, and allow the identification of further research opportunities. This review aims to systematically identify and evaluate research in an attempt to answer the following question, which has been specified using the ‘PECO’ formulation [18]:

- *Population:* As individuals (of any age, gender or ethnicity) develop…
- *Exposure:* …which factors occurring within the family system…
- *(Comparison groups:* Not applicable)
- *Outcome: …*are associated with the reduced likelihood of developing EDs/disordered eating behaviours?

A secondary aim of the review is to investigate the strengths and limitations of research in this field.

## Methods

### Inclusion and exclusion criteria

Papers were included in the review if they met the following criteria:

- The study measures/identifies a factor identified by the authors as potentially ‘protective’.
- A potential protective factor measured/identified occurs at the level of influence of the family system.
- Study outcomes include an appropriate quantitative measure of eating disorder incidence or disordered eating.
- Participants of any age, gender or ethnicity.
- Quantitative study designs.
- Observational designs only: the paper is not investigating the impact of an intervention, treatment or prevention programme.
- Original empirical research only: Not a comment/editorial/review of existing research.
- Papers published or accessible in the English language.
- Research published in peer-review journals.
- Research published in any year up to 2016.

### Information source and search strategy

Studies were identified through electronic searching of the following databases: Psychinfo, Pubmed, Embase, CINAHL and Web of Science Core Collection. The same search terms were used for each database: “eating disorder*” OR “disordered eating” OR “anorexi*” OR “bulimi*” OR “binge eating” OR “binge-eating” OR “EDNOS” OR “ED-NOS”; AND “protective factor” OR “protective” OR “resilienc*”. In addition subject heading searches were included where available: (MeSH term “eating disorders” included for Psychinfo, CINAHL, Embase; MeSH term “protective factors” included for Psychinfo;, MeSH term search unavailable for PubMed or Web of Science). Limits were applied to searches: peer reviewed journal articles, available in the English language. Final searches were conducted on 30^th^ January 2017. The search strategy for Psychinfo is included below as an example:

1. “eating disorder*” OR “disordered eating” OR anorexi* OR bulimi* OR “binge eating” OR binge-eating OR EDNOS OR ED-NOS
2. Subject heading : Eating disorders
3. 1 or 2
4. “protective factor*” OR protective OR resilien*
5. Subject heading: Protective factors
6. 4 or 5
7. 3 and 6
8. LIMIT to journal articles and English language

### Study selection

After removal of duplicates using Mendeley referencing software [19], titles and abstracts were screened to exclude those obviously not relevant to the inclusion criteria. Remaining papers were accessed and read in full, with a two-stage process used to identify papers to include in the final review. At Stage One, papers which included an appropriate quantitative measure of disordered eating and which measured oridentified a potential ‘protective’ factor (as defined by the author) were included. Reviewing reference lists of papers at this stage lead to the identification of additional papers potentially meeting the inclusion criteria. The level of influence of the protective factors measured or identified in each paper was noted in a table, coded as individual/family/peer/school/community/socio-cultural. At Stage Two, only those papers measuring or identifying a protective factor at the level of the family were selected for inclusion in the final review.

### Data collection process and data items

Included papers were read by one researcher to extract relevant data items: was there was a stated intention to identify protective factors, sample size, population, participant characteristics, study design, ED or disordered eating outcome measures used, and key findings related to family factors. These data items were recorded in a form.

The Standard Quality Assessment Criteria (QualSyst) from the Alberta Heritage Foundation for Medical Research was used to appraise study quality, completed by one researcher [20]). This 14 item critical appraisal tool was designed to provide health researchers with a standardised means to assess the quality of studies with varying designs. A final ‘score’ for each study is given by dividing the total score by the total possible score for all applicable items

### Data synthesis

The search strategy and reporting for this systematic review was informed by guidelines from the PRISMA statement [21]. Due to the wide variation of non-randomised study designs, outcomes and methods of analyses used in the studies reviewed, it was not possible to undertake a quantitative synthesis to derive summary statistics. It was decided that a narrative synthesis would be most appropriate to present the key findings and methodological issues identified. Findings from studies using designs which would allow precedence to be established, therefore meeting Kraemer’s criteria for the identification of a protective factor, were considered first [17]. Findings of studies using cross-sectional designs were considered separately

## Results

### Search results

**Fig. 1:**
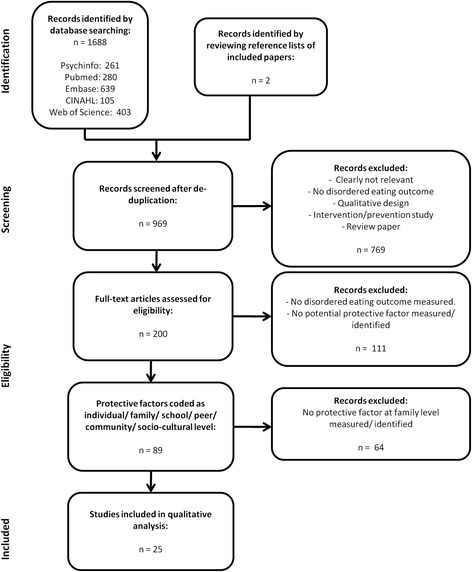
Diagram of systematic search protocol with studies excluded at each stage

969 papers were identified for possible inclusion after de-duplication. Following screening of titles and abstracts, 200 papers which were accessed and read in full to assess eligibility, allowing the identification of 89 papers meeting Stage 1 criteria (including a including an appropriate quantitative measure of disordered eating and the measurement/identification of a factor identified by the authors as potentially ‘protective’). Of these, 25 met Stage 2 criteria (measuring/identifying a protective factor at the level of the family) and were included in the review. Full details of papers included or excluded at each stage of the search protocol can be seen in Fig. 1.

Ten papers made use of designs which would allow precedence to be established. Eight papers made use of longitudinal designs, exploring associations between potential protective and risk factor measures at baseline and eating pathology measures 2- to 5- years later. The majority of these studies used child report to measure family and peer relationship variables, although two studies also included measures completed by parents [22, 23]. Two studies used prospective cohort designs, following a cohort of participants over time to identify cases of EDs and looking for predictors of positive or negative outcomes from earlier time points [24, 25]. Fifteen studies made use of cross-sectional designs, with potential protective factors and disordered eating outcomes measured concurrently. Two of these papers asked adult participants to report retrospectively on aspects of the family environment in their childhood [26, 27]

### Studies with longitudinal and prospective designs

#### Study characteristics

Full details of the participants groups in each study, along with study designs, outcome measures used, and core findings are included in Table 1.

**Table 1:** Participant, sample, design, outcome measures, QualSyst score and key findings for studies with longitudinal and prospective designs

Study	Country	Participant age (years)	Sample size	Participant gender	Participant ethnicity	Sample population	Design	Outcome measure	Qual Syst score	Protective factors identified
Ahren, Chiesa, Koupil, Magnusson, Dalman & Goodman (2013) [24]	Sweden	12 to 23	249894	Mixed (49% female)	Not described	Stockholm Youth Cohort	Prospective cohort study	Cases of ED	1.000	Having full siblings (for females only)
Allen, Gibson, McLean, Davis & Byrne (2014) [22]	Australia	M = 10.78 SD = 1.72	211	Mixed (54% female)	Not described	Childhood Growth and Development Cohort	Longitudinal design (2 years)	ChEDE, CARES (emotional eating)	1.000	Child satisfaction with family life
Berge, Maclehose, Loth, Eisenberg, Bucchianeri, Neumark-Sztainer (2013)* [23]	USA	M = 14.4 SD = 2.0	2348	Mixed (53% female) (Separate analyses)	18.9% White, 29.0% Black, 19.9% Asian, 16.9%Hispanic, 3.7% Native American, 11.6% mixed/other	School students in Project EAT (+parents)	Longitudinal study (2 years)	Dieting/unhealthy or extreme WCBs/binge eating in last year (Yes/No)	.900	Parent discussions around healthy eating. NOT parent discussions about weight
Ferreiro, Seoana & Senra (2012) [34]	Spain	M = 10.84 SD = .78	942	Mixed (49% female) (Separate analyses)	98.5% White	School students	Longitudinal study (4 years)	ChEAT – Spanish Version	.900	Social support (feeling loved and supported by family) (boys only)
Haines, Gillman, Rifas-Shiman, Field & Austin (2009) ^~^ [31]	USA	M = 11.9 SD = 1.6	13448	Mixed (56% female) (Separate analyses)	93% White	Growing Up Today (GUTS) cohort	Longitudinal design (4 years)	Vomiting/use of laxatives/binge eating monthly, dieting weekly (Yes/No)	.900	Family meals
Haines, Kleinman, Rifas-Shiman, Field & Byrn Austin (2010) ^~^ [32]	USA	11 – 18	10540	Mixed (57% female) (Separate analyses)	Not described	Growing Up Today (GUTS) cohort	Longitudinal design (4 years)	Vomiting/use of laxatives/binge eating in past year. Overweight	.850	Family meals
Neumark-Sztainer, Eisenberg, Fulkerson, Story & Larson (2008)* [30]	USA	1/3 M = 12.8 ± 0.8, 2/3 M = 15.8 ± 0.8	2516	Mixed (55% Female) (Separate analyses)	48.5% White, 19.2% Asian, 19.0% African American, 5.8% Hispanic, 3.5% Native American, 3.9% Mixed/Other	School students in Project EAT	Longitudinal design (5 years)	Chronic dieting/unhealthy or extreme WCBs/binge eating in the last year (Yes/No)	.950	Family meals
Neumark-Sztainer, Wall, Story & Sherwood, (2009)* [28]	USA	M = 12.7 ± 0.8	412	Mixed (56% female) (Separate analyses)	45% White, 24% African-American, 16% Hispanic, 6% Asian, 5% Native American, 4% mixed/other	School students in Project EAT identified as overweight	Longitudinal design (5 years)	Extreme WCBs/binge eating in the last year (Yes/No)	.950	Family meals. Family connectedness
Neumark-Sztainer, Wall, Haines, Story, Sherwood & Van der Berg (2007)* [29]	USA	1/3 M = 12.8 ± 0.8, 2/3 M = 15.8 ± 0.8	2380	Mixed (55% female) (Separate analyses)	48.5% White, 19.2% Asian, 19.0% African American, 5.8% Hispanic, 3.5% Native American, 3.9% Mixed/Other	School students in Project EAT	Longitudinal design (5 years)	Extreme WCBs, binge eating in the last year (Yes/No). Overweight.	.850	Family meals
Nicholls & Viner (2009) [25]	UK	30	11211	Mixed	Not described	1970 British Cohort Study	Prospective cohort study	Cases of AN	.950	High maternal BMI. NOT parenting style

*/#/~ = same participant sample

#### Participants and samples

Participants in the studies spanned an age range from 10 to 30 years of age at the time of outcome measurement [22, 25]. Eight of the papers used primarily or exclusively child and/or adolescent participants. Five of the papers used normative samples of school children, (four drawn from the ‘Project EAT’ survey [23, 28–30]), and five papers made use of birth cohorts (two using the same Growing Up Today cohort [31, 32]) Sample sizes ranged from 211 participants to 249,894 participants [22, 24]. The studies were conducted in five different European and North American countries, with six of the papers presenting data on USA-based participant samples. Six of the papers gave information about the ethnicity of participants. The majority of participants reported their ethnicity as White, with the proportion of participants identifying as White varying from 18 to 98% [33, 34]. Seven papers reported analyses separately for females and males.

#### Outcome measures

A range of measures were used to assess disordered eating in the studies. Two studies identified diagnosed cases of ED. Ahren et al. looked at health records for relevant diagnostic codes or evidence of having received treatment for any ED [24]. Nicholls and Viner asked participants if they had ever received a diagnosis of AN [25]. Two studies used standardised measures of disordered eating. One paper used the Child Eating Disorder Examination, a validated clinical interview assessing eating disorder symptoms [35]. One paper used the child version of the Eating Attitude Test a 26-item self report measure assessing dieting, bulimia and food preoccupation, and oral control [36]. The Project Eat survey used by four of the studies included items asking about dieting, binge eating, ‘unhealthy weight control behaviours’ (WCBs; such as skipping meals, eating very little or smoking to control appetite) and ‘extreme WCBs’, (vomiting or use of laxatives, diuretics or diet pills to control weight), as well as asking about current body weight [23, 28–30]. Generally, an indication of ‘Yes’ once or more in the past year to any of these items was taken as evidence of the presence of disordered eating. Note that such individuals may not have met criteria for a full ED. A similar approach was taken by the Growing Up Today cohort survey papers [31, 32]

#### Study quality

Methodological quality was assessed using the QualSyst tool. Total scores for each paper are included in Table 1 (maximum score is 1.000). In general study quality was high, with scores ranging from .850 [29, 32] to 1.000 [22, 24].

All papers described their research questions and objectives sufficiently in their introductory sections. Several did not explicitly state an intention to identify protective factors in addition to risk factors in their introduction, meaning that protective factors were often identified ‘post-hoc’ through the identification of an inverse association [24, 25]. This means that there were few null findings in the literature. All papers were deemed to have designs which were appropriate to answer the study question. Most studies used a method of subject selection designed to obtain an unbiased sample of the relevant target population. The majority of papers included sufficient description of participant characteristics, although a number of papers included less information [25, 32]. All studies used samples of sufficient size to allow adequate statistical power.

All papers used well-defined reproducible outcome measures. Studies using un-validated lists of symptoms rather than standardised measures scored lower. None of these papers included information about the reliability and validity of measures used. Almost all of the studies used self-report measures to assess potential protective factors. Most papers assessed interpersonal factors such family support or connectedness using subjective self-report measures completed by the child.

All papers used appropriate analytic methods, primarily logistic regression analyses. Many papers made multiple comparisons, suggesting a possible risk of Type 1 error. Two of the papers did not include any estimate of variance for their main results [29, 34]. The conclusions of almost all of the studies were well supported by the results. One study draws conclusions about a factor being protective for all adolescents, when in fact this only seems to be the case for overweight adolescents, whose data was analysed separately [23].

#### Results of studies

The papers included in the review identified a number of potential factors in families which may be protective against EDs, along with a range of risk factors.

#### Family composition

Ahren et al.’s prospective analysis of health and other public records for the Stockholm Youth Cohort found that for females only, having a greater number of full siblings was associated with a lower rate of ED by the end of the study period (age 12–27), while the reverse was true for number of half-siblings [24]. In their discussion, they suggest that full siblings might exert a protective effect by ‘diluting’ parental expectations and pressure. They suggested that the finding of the opposite pattern for half-siblings may reflect the fact that these families were more likely to have experienced adverse events such as loss of a parent or divorce.

#### Qualities of family relationships

Several studies looked for associations between the qualities of relationships in families and ED outcomes. Allen et al. conducted a longitudinal study over 2 years following the children of mothers with or without ED history [22]. They found that increases in the child’s reported satisfaction with their family between the ages of 10 and 12 predicted decreases in scores on the ChEDE and loss of control over eating over the 2-year study period. They concluded that being satisfied with family life might be protective against the development of disordered eating in early adolescence, although they did not explore what aspects of family life contributed to a sense of greater satisfaction.

Neumark-Sztainer et al. looked at the impact of family connectedness in overweight children using data from Project EAT [28]. They found that for both boys and girls, greater reported family connectedness at baseline (around age 12) was again associated with reduced likelihood of engaging in binge eating or extreme WCBs 5 years later.

Ferreiro et al. explored the impact of child reported ‘social support’ at age 10 (a composite of feeling loved/supported by family and loved by friends) on the risk of adolescents developing ‘disordered eating’ (scoring above 15 on the ChEAT) over a 5 year period [34]. For both boys and girls, greater depressive symptoms at baseline was predictive of disordered eating 5 years later, but for boys only, support appeared to moderate this effect. Support was also directly predictive of reduced disordered eating at the end of the study for boys only. They concluded that social support, including support from families, may be protective for boys.

#### Family environment around eating and weight

A very large prospective cohort study by Nicholls and Viner looked for associations between a wide range of childhood variables and prevalence of anorexia nervosa by age 30 [25]. Higher maternal BMI at age 10 was found to be associated with reduced risk of AN, as was high self-esteem in childhood. Interestingly, parenting style and the experience of separations from mothers showed no predictive association with disordered eating.

Berge et al. looked at the cross-sectional relationships between the discussions parents were having with their children about healthy eating and weight and the disordered WCBs reported by their children [23]. Many parents were having discussions about healthy eating and/or weight with their children. Overall, parental conversations about healthy eating were associated with the lowest prevalence of disordered WCBs, and parent conversations about weight were associated with higher prevalence. There were slightly different patterns of results for type of behaviour, mothers/fathers and overweight/normal weight children, suggesting healthy eating conversations may only be protective against some behaviours, and more so for overweight than normal weight children.

#### Family meals

Five of the studies investigated the potential protective effect of family meals. Using data from the Project EAT survey, Neumark-Sztainer et al. identified that family meal frequency and a positive atmosphere at meals at baseline was inversely associated with ‘weight related problems’ (extreme WCBs, bingeing and overweight) 5 years later for girls only [29]. Parental weight concern, weight teasing, dieting, weight concern and body dissatisfaction were risk factors for girls developing weight related problems. Another paper in the same group also found that for girls, frequency of family meals at baseline was associated with lower likelihood of engaging in extreme WCBs (but not chronic dieting or binge eating) 5 years later [30]. Interestingly, for boys, regular family meals at baseline were associated with greater likelihood of unhealthy WCBs (non-extreme), in particular skipping meals and eating very little food in meals, 5 years later, but not with other disordered eating outcomes. Family meals may be protective against extreme WCBs for girls. Finally, one of these papers looked at data for a subset of the Project EAT sample who were overweight, and found (in addition to their findings related to family connectedness mentioned earlier) that for girls only family meals with a positive atmosphere at baseline appeared protective against disordered eating 5 years later, as were higher levels of self esteem body satisfaction, and eating regular lunch or dinner [28].

Similar findings are reported by Haines et al. following their analysis of data from the Growing Up Today study [31, 32]. A 2 year longitudinal study found that more frequent family meals in the previous year were associated with significantly lower incidence of purging (for females only), binge eating, and frequent dieting (for females only) [31]. These effects were not modified by age, importance of thinness to parents, frequency of parental comments to child about weight, or maternal dieting behaviours. They concluded that eating family meals frequently may be protective, and this effect is not moderated by negative family interactions around weight and food. A second paper looked at ‘weight related problems’ (bingeing, purging and overweight) in the same dataset and reports that family meal frequency was inversely associated with purging and binge eating for females, both cross-sectionally and longitudinally [32].

### Studies with cross-sectional designs

Fifteen papers made use of cross-sectional designs to identify factors inversely associated with disordered eating outcomes. Full details of the participants groups in each study, along with study designs, outcome measures used, and core findings are included in Table 2.

**Table 2:** Participant, sample, design, outcome measures, QualSyst score and key findings for studies with cross-sectional designs

Study	Country	Participant age (years)	Sample size	Participant gender	Participant ethnicity	Sample population	Design	Outcome measure	Qual Syst score	Protective factors identified
Ackard & Neumark-Sztainer (2001) [26]	USA	M = 20.6, SD = 3.1	560	Female	78.6% White 14.3% Black	University students	Cross sectional design	BULIT-R, EDI −2 (bulimia subscale)	.900	Family meals
Berge, Wall, Larson, Eisenberg, Loth, Neumark-Sztainer (2014)* [33]	USA	M = 14.4 SD = 2.0	2793	Mixed (Separate analyses)	18.9% White, 29.0% Black, 19.9% Asian, 16.9%Hispanic, 3.7% Native American, 11.6% Mixed/Other	School students in Project EAT	Cross sectional design	Dieting/unhealthy or extreme WCBs/binge eating in last year (Yes/No)	.950	Family functioning. Higher sense of connection with either parent. Mothers having knowledge of children’s whereabouts Father’s knowledge of whereabouts (girls only). NOT parental control
Cordero & Israel (2009) [27]	USA	Mode = 19	212	Female	55.9% White, 19.0% Asian/Pacific Islander, 10.9% Latino/Hispanic, 2.8% Black, 1.4% Middle-Eastern, 9.5% Other/Mixed	University students	Cross sectional design	EAT-26	.950	Low negative parental comments about shape and weight
Croll, Neumark-Sztainer, Story & Ireland (2002) [37]	USA	9^th^ and 12^th^ grade students	81247	Mixed (49% female) (Separate analyses)	87% White, 3.5% Asian, 2% Black, 1.5% Hispanic, 1% American Indian.	School students completing Minnesota Student Survey	Cross sectional design	Extreme WCBs/binge eating in last year (Yes/No)	.850	Two parent household. Family connectedness
Fonseca, Ireland & Resnick (2002) [38]	USA	M = 14.4	9042	Mixed (51% female) (Separate analyses)	Not described	School students completing Voice of Connecticut Survey	Cross sectional design	Disordered WCBs (Yes/No)	.950	Family connectedness. Maternal presence in the home. Strong family communication (girls only). High parental expectations (boys only). High supervision and monitoring (girls only- risk factor for boys)
French, Leffert, Story, Neumark-Sztainer, Hannan & Benson (2001)^#^ [16]	USA	6^th^ -12^th^ grade	95395	Mixed (50% female) (Separate analyses)	86% White, 5% multiracial, 4% Hispanic, 2% each African-American, American Indian and Asian.	School students	Cross sectional design	Binge/purge behaviour, weight loss to make others worry ever (Yes/No)	.900	Developmental assets: Family support, Positive family communication, Clear family boundaries
Fulkerson, Story, Mellin, Leffert, Neumark-Sztainer & French (2006) ^#^ [44]	USA	6^th^ -12^th^ grade	99462	Mixed (50% female) (Separate analyses)	86% White, 5% multiracial, 4% Hispanic, 2% each African-American, American Indian and Asian.	School students	Cross sectional design	Binge/purge behaviour, weight loss to make others worry ever (Yes/No)	.900	Family meals
Lampis, Agus & Cacciarru (2014) [48]	Italy	M = 15.9 SD = 1.4	1083	Mixed (55% female)	Not described	School students	Cross sectional design	EDI – Italian version	.900	Family functioning. Mother and father caring style Family cohesiveness.
Loth, Wall, Choi, Bucchianeri, Quick, Larson, Neumark-Sztainer (2015) * [46]	USA	M = 14.5 SD = 1.98	2793	Mixed (53.3% female) (Separate analyses)	18.9% White, 29.0% Black, 19.9% Asian, 16.9% Hispanic, 3.7% Native American, 11.6% mixed/other	School students in Project EAT (+parents)	Cross sectional design	Dieting/unhealthy or extreme WCBs/binge eating in the last year (Yes/No)	.900	Family meals only where: High levels of parent dieting + High enjoyment (boys), Little teasing + Good family functioning + Low levels of weight talk (girls)
McVey, Pepler, Davis, Flett & Abdolell (2002) [39]	Canada	M = 12.9 SD = .62	363	Female	74% White	School students	Cross sectional design	ChEAT	.950	Paternal involvement. Unconditional parental support
Neumark-Sztainer, Wall, Story & Fulkerson (2004)* [43]	USA	M = 14.9 SD = 1.7	4746	Mixed (Separate analyses)	‘Ethnically diverse’	School students in Project EAT	Cross sectional design	Chronic dieting/unhealthy or extreme WCBs/binge eating in the last year (Yes/No)	.950	Family meals
Perkins, Luster & Yank (2002) [40]	USA	M = 14.9 SD = 1.75	18592	Female	83% European American, 8% African American, 3% Native American, 3% Hispanic, 1% Asian or Pacific Islander	Adolescents who have experienced physical abuse	Cross sectional design	Vomiting after eating to control weight two or more times per week.	.950	Family support
Scoffier, Maiano, & D'Arripe-Longueville (2010) [41]	France	M = 15.75 SD = 3.00	227	Female	Not described	Elite aesthetic athletes (dancers/gymnasts/synchronised swimmers)	Cross sectional design	EAT-26 – French Version	.950	Quality of relationship with parents
Twamley & Davis (1999) [42]	USA	M = 20 SD = 2.4	249	Female	77% White	University students	Cross sectional design	EAT-26, BULIT-R combined into composite score	.900	Low family influence to control weight in childhood
Wang et al. (2013) [45]	USA	6^th^ to 8^th^ graders	15461	Mixed (49% female) (separate analyses)	82.3% White, 6.7% Hispanic, 4.3% Black, 4.2% Asian.	School students in Massachusetts Healthy Choices Study	Cross sectional design	Disordered WCBs (Yes/No)	.950	Family meals. Parents providing lifts to physical activity (girls only)

*/#/~ = same participant sample

#### Family composition and qualities of family relationships

In a large study of adolescents in the USA, Croll et al. found that living in a two parent household was cross-sectionally associated with reduced odds of engaging in disordered WCBs, as was and adolescents’ reports of higher family connectedness [37]. They concluded that family connectedness was a significant protective factor. A similar study by Fonseca et al. also found that greater adolescent reported family connectedness was cross-sectionally associated with a reduced likelihood of engaging in disordered WCBs [38]. They also found associations between reduced odds of disordered WCBs and maternal presence in the home for both boys and girls, strong family communication for girls only, and high parental expectations for boys only. There was a different pattern of associations with parental monitoring and supervision for boys and girls, with high supervision and monitoring inversely associated with disordered WCBs for girls, while being associated with increased odds for boys.

Looking at slightly older children (aged 11 to 19) in the USA, Berge et al. found that children’s reports of better ‘family functioning’ were cross-sectionally associated with lower odds of engaging in dieting, binge eating, and a range of unusual and extreme WCBs, as was a reported higher sense of connection with either parent [33]. Mothers generally having knowledge of their children’s whereabouts was associated with fewer disordered eating behaviours for both boys and girls. Fathers’ knowledge of whereabouts was associated with lower odds of disordered WCBs for girls only. Conversely, high reported ‘parental control’ was associated with higher rates of disordered eating, and weakened the protective effect of high family functioning.

French et al. looked for associations of a wide range of ‘developmental assets’ and disordered eating outcomes in adolescents [16]. All of the developmental assets assessed in this very large study using the Profiles of Student Life: Attitudes and Behaviour Survey were cross-sectionally associated with lower rates of disordered WCBs. Family support, positive family communication and clear family boundaries were amongst the strongest ‘discriminating assets’ between those reporting/not-reporting disordered eating.

McVey et al.’s study of early adolescent females looked at unconditional support vs. conditional support, as assessed using the Conditional Support Scale for Parents and the Children’s Perceptions of Parents Scale, and found that unconditional paternal support was cross-sectionally inversely associated with disordered eating assessed using the ChEAT [39]. They found an interaction with negative life events, such that a history of negative life events were less strongly associated with current disordered eating where current unconditional paternal support was high.

A number of studies looked at family factors in adolescents that might be considered ‘at risk’ of developing disordered eating difficulties. Perkins et al. identified female adolescents who had experienced physical abuse [40]. Females reporting physical abuse were more likely to engage in purging behaviour. Current family support was associated with reduced reports of purging in this group, while there was no association between positive family communication and purging. Scoffier et al. studied female adolescents practicing aesthetic sport (dance, gymnastics and synchronised swimming) at an elite level [41]. They found that the quality of parent relationships currently reported on the Self Description Questionnaire was inversely associated with disturbed eating attitudes on the EAT. They suggested that high quality relationships with parents might be protective against the development of disturbed eating attitudes in elite aesthetic athletes.

#### Family environment around eating and weight

Cordero & Israel asked female university students to report retrospectively about factors in earlier life while reporting current disordered eating [27]. Reported lower levels of parental negative comments about weight and shape reported during childhood predicted lower scores on the EAT-26. The strongest predictor of ED symptoms was acceptance of socio-cultural attitudes about appearance. The impact of parental comments was fully mediated by the internalisation of socio-cultural attitudes. They suggested that internalisation of socio-cultural attitudes may act as a pathway for the impact of parental comments on eating pathology. There was no association of parental acceptance or availability with ED symptoms. Twamley and Davis also looked at associations between parents’ comments and influence to control weight and internalisation of thinness norms [42]. In their sample of female undergraduates, awareness of thinness norms was associated with eating pathology, and this effect was mediated by internalisation of thinness norms and body dissatisfaction. Retrospectively reported family influence to control weight moderated the relationship between awareness and internalisation of thinness norms, such that low family influence to control weight in childhood was associated with reduced internalisation of norms in early adulthood, but only where awareness if norms was low. They concluded that low family influence to lose weight may protect against the later development of eating pathology, by buffering against the internalisation of thin-ideals.

#### Family meals

Five studies with cross-sectional designs looked for associations between family meals and disordered eating outcomes. Ackard and Neumark-Sztainer first explored the association between frequency of family dinners in childhood and bulimia symptoms on the BULIT-R and EDI-2 in a sample of female college students [26]. They found that frequency of family dinners while growing up (reported retrospectively) was inversely associated with current bulimic symptoms. The association remained significant after adjusting for other familial factors including family cohesion, independence and achievement orientation. This finding was further supported by a study using data from the Project EAT survey [43]. Increased frequency of family meals, along with prioritising these meals and having a positive meal atmosphere, was shown to be associated cross-sectionally with reduced risk of unhealthy and extreme WCBs for both girls and boys, and with reduced risk of chronic dieting for girls only [43].

Fulkerson et al. used the same dataset as French et al. to explore the association of family meal frequency, disordered eating (bingeing, purging and excessive weight loss) and other high risk behaviours and ‘developmental assets’ [16, 44]. Family meal frequency was cross-sectionally associated with other developmental assets including family support, communication and boundaries and expectations. All high risk behaviours, including disordered eating, were inversely associated with family meal frequency. These associations remained significant even when controlling for family support and communication. They concluded that family meals could be considered a ‘developmental asset’ promoting a range of positive outcomes.

Wang et al. looked at family meal practices and also behaviours around exercise [45]. Adolescents who had family meals most days or every day had decreased odds of disordered WCBs relative to those who never did. Parents’ provision of rides to/from physical activity events was cross-sectionally inversely associated with disordered WCBs for girls. Parental participation in physical activity with children was associated with increased odds of disordered WCBs.

Most recently, Loth et al. have looked again at data from Project EAT [46]. They found that greater frequency of family meals was cross-sectionally associated with lower levels of dieting (girls only), unhealthy WCBs (boys and girls), and extreme WCBs (girls only). There was no association with binge eating. These effects were fairly robust, but a number of interactions were found. For boys, low enjoyment of family meals reversed this association, while high levels of parent dieting were associated with greater association. For girls, family meals were associated with reduced disordered WCBs where there was little teasing, good family functioning, or low levels of weight talk, but this association was reversed where teasing or weight talk were high, or functioning low. They concluded that family meals appear to exert a robust protective effect, but elements of negative mealtime and family weight- related environment may diminish or even reverse this protective effect.

## Discussion

### Key findings

The aim of this review was to identify studies which looked for or identified protective factors against EDs and disordered eating existing in family systems. The papers included in the review make use of a range of designs to explore these associations, and their results highlight a wide range of potential protective factors. Ten papers made use of prospective or longitudinal designs, allowing the identification of potential protective factors according to Kraemer’s definition. These studies looked at family composition, aspects of the quality of family relationships, and at the family environment around eating and weight. Full-siblings appeared to play a potential role in protecting girls against eating disorders [24]. Children’s satisfaction with family life appeared to be protective, as was a reported sense of ‘family connectedness’ [22, 29]. One study identified that social support, including feeling supported by families, was protective against disordered WCBs only for the adolescent boys in their study [34]. Social support, including support from families, is well established as a protective factor promoting general psychological wellbeing. It is thought to play a direct beneficial role over the course of development, while also exerting an indirect effect by ‘buffering’ individuals against the impact of stressful life events [47]. It is perhaps interesting then that there was no association between support and disordered eating outcomes for the girls in Ferreiro et al.’s study. The reasons for this gender difference are unclear, but it is notable that females are more ‘at risk’ of developing difficulties with disordered eating generally. It may be that the protective influence of support is insufficient to overcome the wider socio-cultural pressures promoting eating pathology for females.

The results of studies finding cross-sectional associations provide further clues as to factors which may potentially be protective, although further research would be needed to establish whether these associations are not merely correlations. A range of other family relationship qualities were identified cross-sectionally as being associated with reduced likelihood of disordered eating outcomes: having clear family boundaries, good ‘family functioning’, quality relationships with parents, and unconditional support [16, 33, 37–41, 48]. Many of these factors can be identified as ‘developmental assets’, in that they are factors which have been shown to be related to a wide range of positive developmental outcomes, not just to the absence of eating pathology [3, 16].

This results of papers looking at associations between parenting practices and disordered eating outcomes highlight the importance of careful consideration of nuance and of factors such as gender rather than taking a ‘one-size-fits-all’ approach to studying protective factors, or when using findings such as these to inform prevention efforts [3]. In relation to parenting practices, parents having a caring style was inversely associated with disordered eating, while high levels of ‘parental control’ was associated with increased risk [48]. One study found that parents having a good knowledge of their children’s whereabouts was associated with reduced likelihood of disordered WCBs, while another study found that high levels of parental monitoring and supervision was inversely correlated with disordered WCBs for girls, while in fact being the reverse was true for boys [33, 38]. The only study looking at parenting practices with adult participants, and looking at cases of AN rather than disordered WCBs, found no association between parenting style and AN [25]. They highlight that putative risk and protective factors identified cross-sectionally in childhood do not always show consistent effects when studied longitudinally. This could also reflect the different outcomes measured, with many disordered WCBs reflecting a ‘binge/purge’ type presentation, while AN involves more restriction. It seems likely that different kinds of eating pathology are influenced by different protective and risk factors [49].

Family practices around eating and weight have been studied in some detail. Ten studies, incorporating data from five major surveys, have highlighted a potential protective role of eating regular family meals against developing disordered WCBs. This association has been demonstrated cross sectionally and longitudinally in a range of samples of school children from the USA, most consistently for girls [26, 28–32, 43, 44, 46, 50]. More recently, Loth et al. have looked in more detail at meal frequency in relation to the family environment around food and weight and suggest that while meals are generally associated with reduced odds of engaging in disordered WCBs, in cases where families report high levels of weight talk and teasing and poor family functioning (for girls) or low enjoyment of family meals (for boys) this effect is reversed [46]. Other aspects of the family weight and eating environment identified as protective include high maternal BMI (protective against AN), and having discussions about healthy eating but not about weight (against disordered WCBs) [23, 25]. These results fit well with the findings of qualitative research looking at strategies used by parents to promote positive body image and resilience against disordered eating, such as sensitively filtering communication around body image issues, and promoting positivity around food by shifting the focus of conversations away from body size and weight towards healthy choices and pleasure [51].

### Limitations of the literature

The studies included in the review made use of a range of designs, some of which were more or less suitable for the identification of risk and protective factors. The most commonly used design was a cross-sectional survey, allowing the identification of factors correlated with current disordered WCBs. According to the guidance provided by Kraemer et al., these designs are appropriate to identify ‘correlates’, but these cannot be considered ‘protective’ or ‘risk’ factors unless precedence can be established [17]. Following this guidance, only those studies making use of longitudinal designs could actually be considered to have identified a protective factor. This failure by many studies to make use of appropriate designs for the identification of protective or risk factors severely limits the strength of the conclusions that can be drawn from the literature. They also highlight the important of consideration of time of testing, suggesting that different factors have an influencing role at different times. All of the studies included participants from a range of ages, ignoring the role of time. Nicholls and Viner highlight this in their paper when commenting that risk factors from childhood do not often show consistent associations when measured longitudinally [25]. Going further than this, Kraemer et al. suggest that having identified a protective or risk factor, in order to establish it as causal it is necessary to modify the risk factor and then observe and effect on the outcome of interest [17]. No studies made use of an experimental design which would allow this, and indeed it in unclear in many cases how manipulating variable in this way would be ethical or possible.

There are a number of sources of potential bias in the literature. Many of the papers identified protective factors retrospectively, having set out to measure risk factors and then finding a factor with an inverse association with disordered eating. This post-hoc method of drawing conclusions leaves the literature as a whole at risk of publication bias, as ‘protective’ associations only get mentioned if they have been found. This problem is confounded by the fact that many of the papers assessed high numbers of variables increasing the chance of ‘Type 1’ false positive errors. It is possible therefore that some of the protective relationships identified may be spurious. Support for the existence of a publication bias in this field is evident in the fact that very few papers reported null findings. Where researchers have set out with a specific intention to explore protective factors, the literature is further skewed by the factors that the researchers have chosen to measure. Ten of the papers assessed the same construct, family meal frequency, making this effect seem particularly robust. However looking more closely at the literature, there has been a tendency to write multiple papers using the same population. These papers have also been written exclusively about students in middle or high school in the USA, compromising the generalisability of these findings to other groups.

Regarding clinical utility, some of the papers have assessed factors which could be targeted by interventions, such as family discussions about weight, while other have assessed factors which could be considered more ‘fixed’, such as having full vs. half siblings. Many of the factors assessed, such as ‘good family functioning’ or ‘family connectedness’, are poorly defined and may be highly subjective. Without clearer definition of these factors, and some level of operationalisation of the behaviours which contribute to them, it is hard to know what guidance could be offered to families, if indeed it was practicable to do so. Neither have many of the papers made any attempt to consider the mechanisms by which these factors may exert influence on outcomes, or to explore factors which may mediate or moderate these associations. Consideration of mediating and moderating variables may go some way to elucidating situations where there appear to be different patterns of results, for example where there are differences according to gender or according to type of disordered eating.

### Clinical implications

The identification of protective factors against disordered eating outcomes can inform the development of prevention strategies and interventions [3, 6]. Currently, the vast majority of prevention interventions for EDs work at level of the individual [4, 11]. The findings of these studies could be used to inform prevention approaches at the level of the family. Interventions to address the family environment around eating and weight present one such opportunity, for example supporting families to introduce more frequent, regular family meals and encouraging them to avoid weight related discussions and comments. Even these fairly straightforward seeming interventions would require careful implementation and evaluation, to reduce the risk of iatrogenic effects. For example, it is possible that advising parents to the frequency of family meals without offering guidance and support to reduce weight talk and teasing might be counterproductive and even harmful.

Making full use of the findings of these studies requires a shift in focus from individual pathology to wider social systems. Creative thinking is required to develop ways to intervene at the level of families, communities and policies, with a focus on promoting positive eating and weight related wellbeing outcomes rather than just preventing pathology [15, 52]. Many of the protective factors identified, such as family support, are also protective factors for a range of positive developmental outcomes, meaning effective interventions to promote these factors would be likely to have wide-ranging positive effects [3]. There is a need for a wider scientific and political debate around ways to support families to promote adaptive development, resilience and wellbeing.

### Suggestions for further research

To address the limitations identified above, there is a need for more studies with an explicit objective to investigate protective factors. In many cases ‘correlates’ have been identified through cross sectional designs, presenting opportunities for carefully designed prospective studies to fully test hypotheses about potential protective factors. Further research is also needed to explore the mechanisms and mediating and moderating variables underlying the protective effects identified. These studies could also incorporate data from multiple perspectives rather than relying on an individual’s perception of their relationships with their family. Intervention studies to modify protective factors will allow along the exploration of ‘causality’, and strengthen the clinical utility of these findings. However, such studies are resource intensive and costly.

### Strengths and limitations of review

This review represents an attempt to systematically search and review the literature to create a ‘roadmap’ to a field not previously reviewed. In an attempt to get a comprehensive view inclusion criteria were broad. Papers with cross-sectional designs were included, although these would not allow the identification of protective factors according to Kraemer’s definition [17]. An attempt was made to consider the results of these papers separately from those using designs allowing precedence to be established. Papers in which the outcome ‘disordered eating’ and ‘disordered weight control behaviours’ were included, rather than applying a stricter criteria of cases of eating disorders. This approach means that detailed conclusions about factors influencing different types of eating pathology cannot be drawn [49]. While every effort was made to identify all papers relevant to the topic of the review, it is possible that some papers meeting inclusion criteria may not have been identified. In particular, while reference lists for included studies were scanned for further papers, scanning the reference lists of papers which were excluded was not feasible within the scope of the review.

The search strategy and reporting for this systematic review were informed by guidelines from the PRISMA statement [21]. The wide variation of non-randomised study designs, outcomes and methods of analyses used in the identified studies reviewed meant that it was not possible to undertake a quantitative synthesis to derive summary statistics. This challenge for reviews of this kind is acknowledged in the statement, and in line with the guidance every attempt was made to ensure that the review methods are reported with adequate clarity and transparency to enable readers to critically judge the available evidence and replicate or update the research.

## Conclusions

The aim of this review was to identify and evaluate the current literature regarding protective factors against ED and disordered eating that exist in family systems. The papers discussed investigate a range of potential protective factors including high quality family relationships, and a healthy family environment around eating and weight. The family has a key role to play in protecting young people against disordered eating and supporting adaptive outcomes. Identifying protective factors at this level offers opportunities to understand and possibly prevent these difficulties. Further research into protective factors at this level is essential to inform efforts by clinicians, families, and communities to prevent disordered eating and to promote positive development.

## Abbreviations

ED: Eating disorder
WCB: Weight-control behaviour
